# The Period for Surgical Interference in Acute Intestinal Obstruction

**Published:** 1890-04

**Authors:** B. W. Richardson


### THE PERIOD FOR SURGICAL INTERFERENCE IN ACUTE INTESTINAL OBSTRUCTION.

BY B. W. RICHARDSON, M. D.

—*British Medical Journal*, Feb. 23, 1889.

The conclusions of the author are summarized as follows:

1. That in all cases the use of milder measures, such as purgatives, enemata, and massage, may be safely carried out until the supervention of fæcal vomiting.

2. That as soon as this is established an exploratory incision into the abdomen should be made without delay.

3. That obscurity of diagnosis in presence of this symptom ought not to stand in the way of an operation.

4. That clinical experience has taught that there is very little chance of recovery when once stercoraceous vomiting has begun, unless an operation be performed.

5. That symptoms of collapse are not a contraindication to operative interference.—*Archives Pediatrics.*

